# The Curvilinear Relationship Between Dissatisfaction With the *Status Quo* and Innovative Behavior

**DOI:** 10.3389/fpsyg.2022.849586

**Published:** 2022-03-25

**Authors:** Siyuan Wang

**Affiliations:** Economics and Management School, Wuhan University, Wuhan, China

**Keywords:** dissatisfaction with the *status quo*, innovative behavior, job security, conservation of resources theory, curvilinear relationship

## Abstract

To enhance the understanding the relationship between dissatisfaction with the *status quo* and innovation, this study proposed that dissatisfaction with the *status quo* has a curvilinear relationship with innovative behavior and job security moderates the association between these two variables. An investigation based on 214 employees from Chinese companies was conducted. The results indicated that dissatisfaction with the *status quo* has an inverted U-shaped relationship with idea dissemination and idea implementation, and job security moderates the inverted U-shaped relationship. Specifically, for individuals with a low job security, the curvilinear relationship is stronger, whereas for individuals with a high job security, the slope of the curve becomes nearly flat, thus losing the inverted-U effect. Theoretical and practical implications are discussed, and directions for future research are outlined.

## Introduction

With the rapid development of economy and society, the continuous progress of science and technology, and the increasing intensification of global competition, enterprises must rely on a steady stream of innovation to produce services and products that meet the needs of consumers in order to win the core competitive advantage (Scott and Bruce, 1994; Anderson et al., 2014; Zhang et al., 2018; Purc and Laguna, 2019; Li et al., 2020; Guo et al., 2022; Saura et al., 2022). Especially in today’s changeable and complex business environment, innovation is the key to the survival and development of an organization (Scott and Bruce, 1994; Sacramento et al., 2013; Yuan and Woodman, 2021). Employees are the valuable resources of the organization and the initiator of the creativity of all products or services. Therefore, how to stimulate employees’ creativity and promote employees’ innovative behavior has become an important topic in academia and business circles for a long time (Perry-Smith and Shalley, 2003; Liu et al., 2017; Lee and Trimi, 2021). Based on this, this study intends to explore what factors can promote individual innovative behavior.

This study focuses on the factor of dissatisfaction with the *status quo*. Generally speaking, job dissatisfaction will damage organizational performance. However, from the perspective of organizational innovation, job dissatisfaction may have a positive effect (Yuan and Woodman, 2010). The existing literature on innovative behavior shows that dissatisfaction is an important factor causing employees’ innovation behavior (Zhou and George, 2001; Birkinshaw and Mol, 2006; Yuan and Woodman, 2010). Birkinshaw and Mol (2006) used case studies to find that the first step of innovation is dissatisfaction with the *status quo*. What’s more, using the method of questionnaire survey, some other researchers found that when employees are dissatisfied with the current situation, they are likely to engage in innovative behavior (Zhou and George, 2001; Yuan and Woodman, 2010). However, both in theory and practice, job dissatisfaction is often associated with some negative factors related to organizational performance (De Clercq et al., 2021), such as employee’s job dissatisfaction diminishes their organizational commitment (Kim and Back, 2012) or job performance (Rayton and Yalabik, 2014). The purpose of this study is to explore the real relationship between dissatisfaction with the *status quo* and innovative behavior and the possible boundary condition.

This study has the following two important implications. First, this make a contribution to the dissatisfaction with the *status quo* and innovation literature. On the one hand, examining employee innovative behavior from the perspective of dissatisfaction with the *status quo* extends current knowledge on the influence of job dissatisfaction on employee innovation (Zhou and George, 2001; Birkinshaw and Mol, 2006; Yuan and Woodman, 2010). On the other hand, prior research has employed a linear framework when examining the effect of dissatisfaction with the *status quo* on employee innovation and obtained inconsistent results, which have puzzled scholars to some extent. By considering an inverted U-shaped relationship between dissatisfaction with the *status quo* and innovative behavior, this study may help to explain the initially inconsistent effects of dissatisfaction with the *status quo* on employee innovative behavior observed in previous research. Second, to deepen the understanding of the potential boundary conditions related to this non-monotonic association, this study examine the moderating role of the job security in the curvilinear dissatisfaction with the *status quo* on employee innovative behavior relation. This study theorizes that the job security diminishes the likelihood that an employee will fall prey to the potentially debilitating negative consequences of dissatisfaction with the *status quo*. This interaction analysis offers important insight into the effect of dissatisfaction with the *status quo* related constructs on employee innovative behavior.

## Literature Review and Hypothesis

### Conservation of Resources Theory

Conservation of resources (COR) theory is essentially a motivation theory model, which holds that individuals have the motivation to acquire, preserve and develop important resources (Hobfoll, 1989). In the early stage, it was mainly used to explain the nature of stress and the coping strategies when individuals face stressors (Hobfoll et al., 2018). For more than 20 years, COR theory has been continuously developed and widely used in the research fields of work family conflict, emotional exhaustion, and organizational citizenship behavior (Halbesleben et al., 2014; Astakhova, 2015; Anjum et al., 2020; Trzebiatowski and Triana, 2020; Hsieh et al., 2021). COR theory divides resources into four categories: material resources, conditional resources, self-resources and energy resources (Hobfoll, 1989). Among them, material resources refer to the resources that determine individual socio-economic status, such as cars, housing and labor tools. Conditional resources refer to resources that can create conditions for individuals to obtain key resources, such as friends, marriage and power. Self-resources refer to individual personality characteristics, such as psychological capital, self-efficacy and self-esteem. Energy resources refer to resources that help individuals obtain other resources, such as time, money and knowledge.

Conservation of resources theory also contains four basic principles, namely, the primacy principle of resource loss, the principle of resource investment, the spiral principle of resource loss and the spiral principle of resource acquisition (Hobfoll et al., 2018). Among them, the primacy principle of resource loss is that resource loss is more important than resource acquisition. The principle of resource investment is that in order to prevent resource loss or recover and obtain resources from resource loss, individuals need to invest and develop resources. The spiral principle of resource loss is that individuals with fewer resources are more likely to suffer resource loss. The spiral principle of resource acquisition means that individuals with more resources are more likely to look for opportunities, take risks and invest resources in order to obtain more resources.

In this study, dissatisfaction with the *status quo* is a stressor, which can stimulate employees’ tendency of resource acquisition and resource loss at the same time. Specifically, on the one hand, when a certain degree of dissatisfaction will stimulate individuals to use the resource investment principle to obtain new resources by participating in innovative behavior. On the other hand, excessive dissatisfaction will lead to the desperate situation of resources, so as to avoid resource loss by reducing innovation behavior. Therefore, this study uses COR theory to explain the inverted U-shaped relationship between dissatisfaction with the *status quo* and innovation behavior. What’s more, job security can be used as a resource supplement process to make up for the loss of employee’s resources. Therefore, this study will further explore the moderating role of job security.

### Dissatisfaction With the *Status Quo* and Innovative Behavior

This study intends to explore the inverted U-shaped relationship between dissatisfaction with the *status quo* and innovation behavior. First, in the face of mild dissatisfaction with the *status quo*, employees can realize that their organization has failed and can’t achieve performance goals. At this time, employees may solve these problems in some ways and make other members aware of the causes of the problems (De Clercq et al., 2021), so as to jointly help the organization improve this negative situation and participate in innovation behavior. In addition, employee innovation is often the original starting point of organizational innovation, that is, employee innovation is an important form for employees to express job dissatisfaction. The research done by Frohman (1997) describes two typical behaviors of expressing job dissatisfaction identified by participants, including “proposing new ways to solve problems” and “putting forward suggestions on how to improve things,” and these two behaviors themselves are consistent with employee innovation commonly defined by people. Therefore, appropriate expression and behavior response will not only effectively release employee’s dissatisfaction, but also encourage participatory and creative activities for improvement. It can not only introduce employee’s job dissatisfaction into a positive channel eager for change, but also help employees improve their work and make continuous progress (Kluger and DeNisi, 1996; Oldham and Cummings, 1996).

Second, when the dissatisfaction with the *status quo* exceeds the inflection point, that is, when they are very dissatisfied with the *status quo* of the organization, employees will face a resource desperate situation (De Clercq et al., 2021), in which case employee’s innovative behavior will be reduced. On the one hand, employees in resource desperate situations do not have sufficient resources to engage in innovative behavior. Innovation enables individuals to perform some new tasks and processes at work (Gonzalez-Roma and Hernandez, 2016), and increased personal workload (Anderson et al., 2014), which then led to greater tension and anxiety (Gonzalez-Roma and Hernandez, 2016). These all require individuals to invest more resources to deal with these activities. On the other hand, individuals trapped in a resource dilemma start a defense mechanism of self-protection (Hobfoll et al., 2018). In order to avoid further resource loss, they will no longer participate in innovative behaviors that may consume resources. Based on this, this study puts forward the following research hypotheses:

Hypothesis 1: Dissatisfaction with the *status quo* has an inverted U-shaped relationship with idea generation (1a), idea dissemination (1b) and idea implementation (1c).

### The Moderating Role of the Job Security

Previous researches showed that employees usually have different behavioral responses when facing job dissatisfaction (Zhou and George, 2001). This study further analyzes the boundary condition between dissatisfaction with the *status quo* and innovative behavior. As the core dimension of high-performance human resources practice (Sun et al., 2007), job security refers to an employee’s expectations about the stability and longevity of his or her job in an organization (Lu et al., 2017). Innovation activities have certain risks, so organizations need to provide a relatively safe environment (Anderson et al., 2014). Previous studies on job security focused on its impact on organizational identity (Ma et al., 2016), organizational satisfaction (Gholamreza et al., 2011) and job performance (Kraimer et al., 2005), ignoring the relationship between job security and innovation. Based on the COR theory (Hobfoll, 1989; Hobfoll et al., 2018), this study believes that job security can be used as a resource supplement process to make up for the loss of employee’s resources. Therefore, this study will further explore the moderating role of job security.

On the one hand, employees with high job security are more confident that they will continue to stay in the current organization (Witte, 1999; Ma et al., 2016). For example, previous studies have found that individuals who perceive a high level of job security have a high sense of organizational identity and believe that the organization is very concerned about their career development (Ma et al., 2016). Employees with a high sense of job security will have a higher sense of organizational identity. Even if they are dissatisfied with the current situation, they will not have the motivation to change the current situation. As a resource supplement, high job security can make up for the loss caused by dissatisfaction with the *status quo*. Therefore, in the situation of high job security, employee’s innovative behavior will not be affected by dissatisfaction with the current situation.

On the other hand, employees with low job security feel more risks and uncertainties in their future employment (Loi et al., 2011) and experience more psychological pressure. For example, previous researcher proved that job insecurity has been regarded as a major stressor and may lead to negative stress reactions in the workplace (Witte, 1999; Loi et al., 2011). When employees have a low sense of job security and face a certain degree of dissatisfaction with the *status quo*, they will have the internal motivation to change the current situation, so as to encourage them to produce innovative behavior. Because participation in innovation activities can obtain new resources to a certain extent. In addition, when employees with low sense of job security face excessive dissatisfaction with the *status quo* at the same time, employees will be in a resource dilemma. At this time, they have no motivation to obtain new resources, so their innovation behavior will be reduced. Therefore, this study believes that under the situation of low job security, the inverted U-shaped relationship between dissatisfaction with *status quo* and innovation behavior will be stronger.

Based on this, this study puts forward the following research hypotheses:

Hypothesis 2: Job security moderates the inverted U-shaped relationship between dissatisfaction with the *status quo* and individual innovative behavior (idea generation (1a), idea dissemination (1b) and idea implementation (1c), such that the curvilinear relationship is stronger for individuals with a low job security.

**FIGURE 1:**
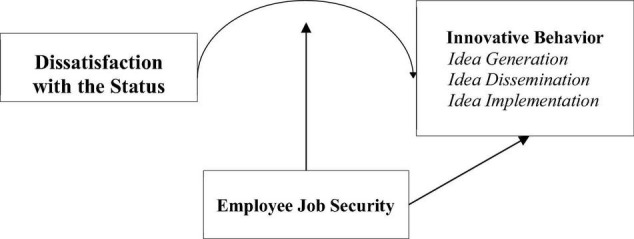
Research model.

The research model is shown in Figure 1.

## Methods and Results

### Participants and Procedures

The samples of this study were taken from five information technology companies in China, which located in Beijing, Guangzhou, Shenzhen, Chengdu and Chongqing. In order to reduce common method variance (Podsakoff et al., 2003), this study adopted multi-time points and multi-source design. In the first-wave survey (T1), this study sent questionnaires to 350 of employees (response rate as 76.57%). This study surveyed employee dissatisfaction with the *status quo* and demographic variables. And one month after the first survey, the second questionnaire (T2) was distributed to the 268 employees and their leaders. This study surveyed the employee job security and innovative behavior, and 214 of whom returned complete questionnaires (response rate as 79.85%), constituting the final sample of this study. Of the 214 employees in the sample, 64.95% were male, the average age was 30.42 years (*SD* = 5.07), the average tenure was 6.65 years (*SD* = 4.92), 95.79% had received a bachelor’s or higher degree.

### Measures

This study administered the survey in Chinese, following the commonly used translation and back-translation procedure in which the scales were translated from English into Chinese by one professor (Brislin, 1980). This study measured all items on a 7-point Likert scale (1 = strongly disagree, 7 = strongly agree).

#### Dissatisfaction With the *Status Quo*

Employees rated their dissatisfaction at T1 using the three-item scale developed by Yuan and Woodman (2010). An example item is “Many things in my department need improvement” (α = 0.88).

#### Job Security

Employees rated their job security at T2 using the two-item scale developed by Sun et al. (2007). An example item is “I can be expected to stay with this organization for as long as I wish” (α = 0.76).

#### Innovative Behavior

Leaders rated their employee’s innovative behavior at T2 using the nine-item scale created by Janssen (2000). An example item for idea generation is “I create new ideas for improvements.” (α = 0.85), for idea dissemination is “I make important organizational members enthusiastic for innovative ideas.” (α = 0.90), and for idea implementation is “I transform innovative ideas into useful applications” (α = 0.92).

#### Control Variable

As suggested by previous researches (Ng and Lucianetti, 2016), this study controlled for employee age (in years), gender (1 = male, 2 = female), education (1 = vocational school/college and below, 2 = bachelor degree, 3 = master degree, 4 = doctor degree) and tenure (in years), as prior research suggested that these variables may affect employees’ willingness to engage in innovative activities.

### Analytical Approach

Following prior works (Haan et al., 2015; Lin et al., 2017; Zhang and Zhou, 2019), this study used regression analysis with quadratic terms to test the inverted U-shaped relationship between employee dissatisfaction with the *status quo* (DSQ) and innovative behavior (IB) in Hypothesis 1:


(1) IB=γ+00γ(controls)c0+γ(DSQ)10+γ(DSQsquared)20

The γ_20_ had to be negative and significant to indicate the presence of an inverted U-shaped relationship between employee dissatisfaction with the *status quo* and innovative behavior. Next, this study used the following equation to test the moderating effect of job security (JS) proposed in Hypothesis 2:


(2) IB=γ+00γ(controls)c0+γ(DSQ)10+γ(DSQsquared)20

+γ(JS)30+γ(DSQ×JS)40+γ(DSQsquared×JS)50

In Equation (2), significance for γ_50_ would indicate that the inverted U-shaped relationship between employee dissatisfaction with the *status quo* and innovative behavior would vary as a function of job security.

## Results

### Confirmatory Factor Analyses

This study conducted confirmatory factor analyses (CFAs) to examine the distinctiveness all the variables (dissatisfaction with the *status quo*, job security, idea generation, idea dissemination, and idea implementation). Table 1 shows the results of model fit comparisons. The hypothesized five-factor model showed satisfactory fit (χ^2^(67) = 86.75, CFI = 0.99, TLI = 0.98, RMSEA = 0.04) and had better fit than all of the alternative models.

**TABLE 1:** Model fit results for confirmatory factor analyses.

Models	χ^2^	*df*	RMSEA	CFI	TLI
Five-factor model	86.75	67	0.04	0.99	0.98
Four-factor model^a^	172.46	71	0.08	0.95	0.93
Three-factor model ^b^	341.203	74	0.13	0.86	0.83
Two-factor model ^c^	416.98	76	0.14	0.83	0.79
Single-factor model ^d^	739.77	77	0.20	0.66	0.60

*n = 214.*

*^a^Four-factor model: idea dissemination and implementation are combined.*

*^b^Three-factor model: idea generation, dissemination and implementation are combined.*

*^c^Two-factor model: job security, idea generation, dissemination and implementation are combined.*

*^d^One-factor model: All variables are combined.*

### Correlations

The means, standard deviations, and correlations among the research variables are presented in Table 2. Employee dissatisfaction with the *status quo* was positively correlated with idea generation (*r* = 0.28, *p* < 0.01), idea dissemination (*r* = 0.32, *p* < 0.01), and idea implementation (*r* = 0.33, *p* < 0.01). Job security was also positively correlated with idea generation (*r* = 0.33, *p* < 0.01), idea dissemination (*r* = 0.34, *p* < 0.01), and idea implementation (*r* = 0.39, *p* < 0.01).

**TABLE 2:** All variables’ means, standard deviations, and correlation matrix.

Variables	M	SD	1	2	3	4	5	6	7	8
(1). Gender	1.35	0.48								
(2). Age	30.42	5.07	−0.14*							
(3). Education	2.15	0.53	0.03	0.34**						
(4). Tenure	6.65	4.92	−0.11	0.91**	0.18**					
(5). DSQ	4.82	1.24	0.13	−0.06	0.05	0.01				
(6). Job Security	4.61	1.02	0.10	−0.04	−0.01	−0.02	0.17*			
(7). Idea Generation	4.69	1.25	−0.02	0.05	0.06	0.08	0.28**	0.33**		
(8). Idea Dissemination	4.59	1.36	0.07	−0.03	−0.02	0.04	0.32**	0.34**	0.55**	
(9). Idea Implementation	4.63	1.33	0.11	−0.06	−0.05	0.03	0.33**	0.39**	0.49**	0.78**

n = 214, **p < 0.01 *p < 0.05. DSQ: dissatisfaction with the status quo.

### Hypothesis Testing

Table 3 shows that the curvilinear relationship proposed by hypothesis 1. As for the idea generation, the squared term was not statistically significant (−0.03, *ns*, M_2_, R^2^ = 0.09). Therefore, the Hypothesis 1a was not supported.

**TABLE 3:** Results of hierarchical linear modeling-based regression analysis.

Variable	Idea Generation		Idea Dissemination		Idea Implementation	
	M_1_	M_2_	M_3_	M_4_	M_5_	M_6_
Constant	5.81**	5.19**	7.06**	6.18**	7.38**	6.53**
Gender	–0.08	–0.16	0.14	0.03	0.25	0.14
Age	–0.07	–0.02	−0.12*	–0.06	–0.14	–0.07
Education	0.22	0.12	0.12	–0.02	0.06	–0.08
Tenure	0.08	0.04	0.13	0.06	0.14	0.07
Employee DSQ		0.25**		0.16*		0.19*
Employee DSQ^2^		–0.03		−0.16**		−0.14**
R^2^	0.02	0.09	0.04	0.20	0.05	0.19
F value	0.98	3.60**	1.98	8.44**	2.93*	8.35**

n = 214, ** p < 0.01 * p < 0.05. DSQ: dissatisfaction with the status quo.

**FIGURE 2:**
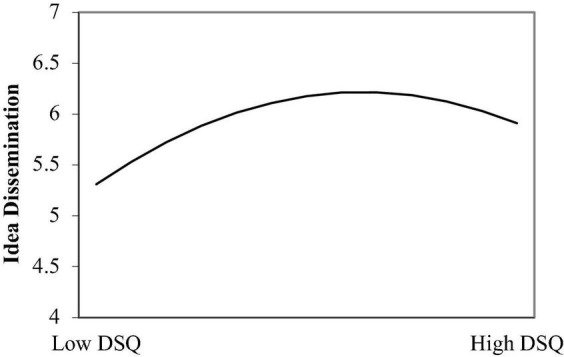
The Inverted U-shaped relationship between dissatisfaction with the *status quo* (DSQ) and idea dissemination.

As for the idea dissemination, the squared term was statistically significant (−0.16, *p* < 0.01, M_4_, R^2^ = 0.20; see the curve in Figure 2). This study also calculated the inflection point and found that the inflection point of dissatisfaction with the *status quo* was 0.49 (95%_CI_ = [0.017; 1.418]; the data was grand-mean centered). If dissatisfaction with the *status quo* was lower than 0.49, the trend of the relation with idea dissemination was upward (slope = 1.41, *p* < 0.01). It turned downward when dissatisfaction with the *status quo* was larger than 0.49 (slope = −0.55, *p* < 0.01). Using a t-test, this study found significant differences between the slopes of the simple main effects before and after the inflection point (*t* = 2.70, *p* < 0.01). The Hypothesis 1b was supported.

**FIGURE 3:**
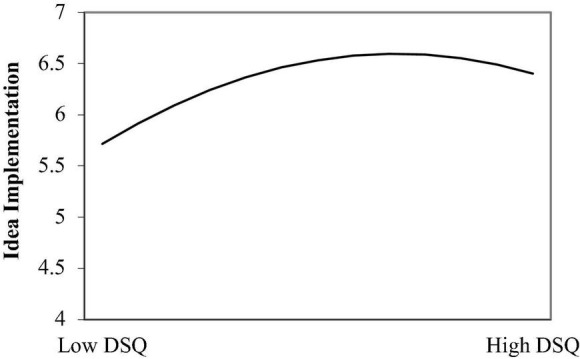
The Inverted U-shaped relationship between dissatisfaction with the *status quo* (DSQ) and idea implementation.

As for the idea implementation, the squared term was statistically significant (−0.14, *p* < 0.01, M_6_, R^2^ = 0.19; see the curve in Figure 3). This study also calculated the inflection point and found that the inflection point of dissatisfaction with the *status quo* was 0.68 (95%_CI_ = [0.102; 2.053]; the data was grand-mean centered). If dissatisfaction with the *status quo* was lower than 0.68, the trend of the relation with idea dissemination was upward (slope = 1.24, *p* < 0.01). It turned downward when dissatisfaction with the *status quo* was larger than 0.68 (slope = −0.41, *p* < 0.05). Using a t-test, this study found significant differences between the slopes of the simple main effects before and after the inflection point (*t* = 2.06, *p* < 0.05). The Hypothesis 1c was supported.

Table 4 shows that the moderating effect proposed by hypothesis 2. As for the idea generation, the positive relationship between job security and idea generation was statistically significant (0.40, *p* < 0.01, M_7_, R^2^ = 0.12). However, the results indicated that job security non-significantly interacted with dissatisfaction with the *status quo* squared to influence idea generation (−0.08, *ns*, M_8_, R^2^ = 0.19). Therefore, the Hypothesis 2a was not supported.

**TABLE 4:** Results of hierarchical linear modeling-based regression analysis.

Variable	Idea Generation		Idea Dissemination		Idea Implementation	
	M_7_	M_8_	M_9_	M_10_	M_11_	M_12_
Constant	5.65**	5.19**	6.88**	6.07**	7.18**	6.48**
Gender	–0.16	–0.22	0.06	–0.03	0.15	0.06
Age	–0.05	–0.02	−0.11*	–0.05	−0.12**	–0.07
Education	0.20	0.07	0.11	0.05	0.04	0.01
Tenure	0.07	0.03	0.12*	0.05	0.12**	0.07
Employee DSQ	0.40**	0.21**	0.44**	0.11	0.49**	0.12
Employee DSQ^2^		–0.04		−0.14**		−0.11**
Job Security (JS)		0.47**		0.23*		0.29**
DSQ * JS		–0.01		0.01		–0.05
DSQ^2^ * JS		–0.08		0.10*		0.11*
R^2^	0.12	0.19	0.14	0.29	0.19	0.32
*F* value	5.91**	5.25**	7.03**	9.37**	9.87**	10.88**

n = 214, **p < 0.01 *p < 0.05. DSQ: dissatisfaction with the status quo.

**FIGURE 4:**
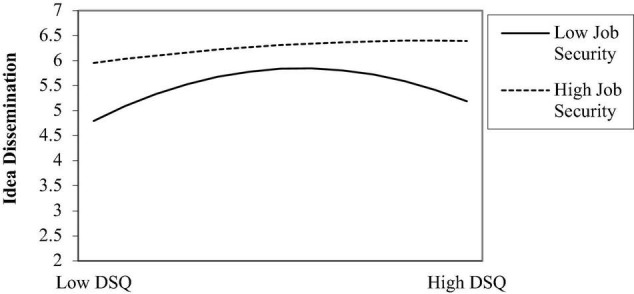
Relationship between dissatisfaction with the *status quo* (DSQ) and idea dissemination as a function of job security.

As for the idea dissemination, the positive relationship between job security and idea dissemination was statistically significant (0.44, *p* < 0.01, M_9_, R^2^ = 0.14). What’s more, the results also indicated that job security significantly interacted with dissatisfaction with the *status quo* squared to influence idea dissemination (0.10, *p* < 0.05, M_10_, R^2^ = 0.29; see the curve in Figure 4). Therefore, the Hypothesis 2b was supported.

**FIGURE 5:**
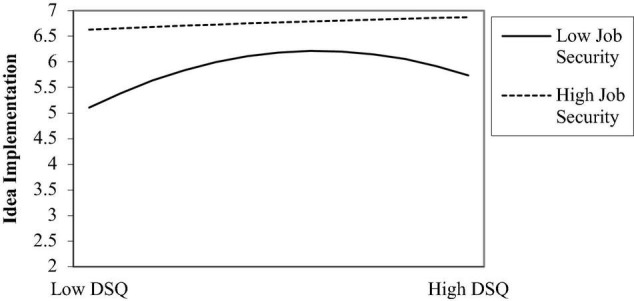
Relationship between dissatisfaction with the *status quo* (DSQ) and idea implementation as a function of job security.

As for the idea implementation, the positive relationship between job security and idea implementation was statistically significant (0.49, *p* < 0.01, M_11_, R^2^ = 0.19). What’s more, the results also indicated that job security significantly interacted with dissatisfaction with the *status quo* squared to influence idea implementation (0.11, *p* < 0.05, M_12_, R^2^ = 0.32; see the curve in Figure 5). Therefore, the Hypothesis 2c was supported.

## Discussion

The purpose of this study was to examine the relationship between dissatisfaction with the *status quo* and employee innovative behavior. Specifically, this study examined whether the relationship between dissatisfaction with the *status quo* and employee innovative behavior could be characterized by an inverted U-shape function and whether the job security moderates this relationship. The results suggest that the relationship between dissatisfaction with the *status quo* and employee innovative behavior (idea dissemination and idea implementation) is non-monotonic and that an inflection point indeed exists. Furthermore, in accordance with the hypothesis, this study found that the job security moderates the squared dissatisfaction with the *status quo* term for idea dissemination and idea implementation, with dissatisfaction with the *status quo* exerting a stronger effect on employee innovative behavior in employees with a low job security. By contrast, for employees with a high job security, the slope of the curve became nearly flat, thus indicating the lack of inverted-U effect. Therefore, this study has made some contributions in both theory and practice.

In addition, hypotheses 1a and 2a were not supported in this study. Based on the COR theory, this study believes that employees are unlikely to invest too much time to generate new ideas when they are dissatisfied with the *status quo*, but they will directly participate in the idea dissemination and implementation. Idea dissemination and implementation are more conducive to resource recovery and obtain more new resources, so as to make up for the resource loss caused by the dissatisfaction with the *status quo*.

### Theoretical Implications

First, and perhaps most importantly, this study demonstrates that dissatisfaction with the *status quo* has a curvilinear relationship with employee innovative behavior (idea dissemination and idea implementation). Dissatisfaction with the *status quo* has received less attention in previous studies, but it is very important for innovation (Yuan and Woodman, 2010; Kim and Back, 2012; De Clercq et al., 2021). Previous studies have found that there is a linear relationship between dissatisfaction with the *status quo* and innovative behavior (Yuan and Woodman, 2010), that is, dissatisfaction with the *status quo* can positively affect innovation (Zhou and George, 2001; Birkinshaw and Mol, 2006; Yuan and Woodman, 2010). However, some studies have found that dissatisfaction with the *status quo* will bring negative results (Kim and Back, 2012; De Clercq et al., 2021). This study combined these two parts of literature and found that focusing on a linear relationship will not capture the essence of dissatisfaction with the *status quo*; instead, a more complex understanding of how dissatisfaction with the *status quo* influences employee outcomes should be developed. Therefore, these findings advance the understanding of the influence of dissatisfaction with the *status quo* on innovative behavior and enrich the literature on the construct of dissatisfaction with the *status quo*.

Second, in an effort to derive a contingent condition of the curvilinear relationship, this study examined the moderating effect of the job security on this relationship. Based on COR theory (Hobfoll, 1989; Hobfoll et al., 2018), according to the resource perspective in previous studies (De Clercq et al., 2016), this study found that the job security moderates the curvilinear relationship between dissatisfaction with the *status quo* and employee innovative behavior. Furthermore, consistent with prior findings, this study found that dissatisfaction with the *status quo* has a stronger effect on innovative behavior for employees with a low job security. The difference between our results and those of prior studies (Yuan and Woodman, 2010; Kim and Back, 2012; De Clercq et al., 2021) is that this study found that the association between dissatisfaction with the *status quo* and employee innovative behavior can be described by an inverted-U shape. This study further found that in teams with a high job security, the slope of the curve became nearly flat, thus losing the inverted-U effect. An explanation for this result may lie in the buffering effect of the job security. Employees with a high job security are not sensitive to dissatisfaction with the *status quo*. This suggests that dissatisfaction with the *status quo* is not necessarily a concern if job security is high. The analysis of the quadratic term interaction offers important insight into the effect of dissatisfaction with the *status quo* on employee innovative behavior and takes a step toward resolving the disputes concerning the interaction effect of dissatisfaction with the *status quo* on employee innovative behavior. Therefore, this study expands the previous research on the boundary conditions of innovation behavior (Saura et al., 2022).

### Practical Implications

First, the curvilinear effect of dissatisfaction with the *status quo* suggests that dissatisfaction with the *status quo* may be a double-edged sword, which must be handled with care. Such dissatisfaction with the *status quo* can help organizations to enhance employee innovative behavior. However, according to the findings of this study, dissatisfaction with the *status quo* and employee innovative behavior are related in a curvilinear fashion, and thus organizations should be cautious of high dissatisfaction with the *status quo*. As the proverbs state, “too much can be worse than too little” and “everything in moderation; nothing in excess” (Li et al., 2016). Therefore, for some employees who are not satisfied with the *status quo*, the organization can take some measures to improve their satisfaction. For example, an organization can regularly investigate employees’ satisfaction and the feedback on the organization, and arrange special personnel to solve these problems.

Second, by examining the boundary condition and contextual effect underlying the negative consequences of otherwise beneficial states such as dissatisfaction with the *status quo*, our study can inform and help practitioners understand how to mitigate the negative consequences of dissatisfaction with the *status quo*. The current research suggests that the job security has a moderating effect on the dissatisfaction with the *status quo*-employee innovative behavior association. Therefore, organizations should improve employees job security. For example, some cultural exchange activities are held to strengthen employees’ perception of corporate culture, so as to improve their sense of belonging, significance and security to the organizations.

### Limitations and Future Research

The current study has several limitations that indicate directions for future research. First, the study used a cross-sectional design, and thus this study cannot draw strong causal inferences. Future research might employ longitudinal designs, whereby measures of dissatisfaction with the *status quo* and innovative behavior are collected over several periods to permit an examination of the causal relationship between dissatisfaction with the *status quo* and employee innovative behavior. Second, this study has demonstrated that a moderate level of job security is optimal for employee innovative behavior. In order to provide more useful guidance to practitioners, future research can focus on exploring more moderators. Third, this study didn’t explore the mediating mechanism of dissatisfaction with the *status quo* affecting employee innovative behavior. Future researchers can make a more in-depth discussion based on different theoretical perspectives. Fourth, this study only selects employees in technology companies as the research object. However, different types of companies may have some differences. In order to make the research results more universal, employees in other industries can be selected as the research object in future research. In addition, future research can increase the sample size as much as possible to reduce the sample error, and future research can also add some control variables, such as the salary range and the position in the company.

## Data Availability Statement

The original contributions presented in the study are included in the article/supplementary material, further inquiries can be directed to the corresponding author/s.

## Ethics Statement

Ethical review and approval was not required for the study on human participants in accordance with the local legislation and institutional requirements. The patients/participants provided their written informed consent to participate in this study.

## Author Contributions

SW contributed to designing, analyzing, and writing the study.

## Conflict of Interest

The author declares that the research was conducted in the absence of any commercial or financial relationships that could be construed as a potential conflict of interest.

## Publisher’s Note

All claims expressed in this article are solely those of the authors and do not necessarily represent those of their affiliated organizations, or those of the publisher, the editors and the reviewers. Any product that may be evaluated in this article, or claim that may be made by its manufacturer, is not guaranteed or endorsed by the publisher.
